# Atg8 family proteins, LIR/AIM motifs and other interaction modes

**DOI:** 10.1080/27694127.2023.2188523

**Published:** 2023-03-19

**Authors:** Vladimir V. Rogov, Ioannis P. Nezis, Panagiotis Tsapras, Hong Zhang, Yasin Dagdas, Nobuo N. Noda, Hitoshi Nakatogawa, Martina Wirth, Stephane Mouilleron, David G. McEwan, Christian Behrends, Vojo Deretic, Zvulun Elazar, Sharon A. Tooze, Ivan Dikic, Trond Lamark, Terje Johansen

**Affiliations:** a Institute for Pharmaceutical Chemistry, Department of Biochemistry, Chemistry and Pharmacy, Goethe University, 60438 Frankfurt, am Main, and Structural Genomics Consortium, Buchmann Institute for Molecular Life Sciences, Goethe University, 60438 Frankfurt am Main, Germany; b School of Life Sciences, University of Warwick, CV4 7AL Coventry, UK; c National Laboratory of Biomacromolecules, CAS Center for Excellence in Biomacromolecules, Institute of Biophysics, Chinese Academy of Sciences, Beijing, China and College of Life Sciences, University of Chinese Academy of Sciences, Beijing, China; d Gregor Mendel Institute, Austrian Academy of Sciences, Vienna BioCenter, Vienna, Austria; e Institute for Genetic Medicine, Hokkaido University, Kita 15, Nishi 7, Kita-ku, Sapporo 060-0815, Japan; f School of Life Science and Technology, Tokyo Institute of Technology, Yokohama, Japan; g Molecular Cell Biology of Autophagy, The Francis Crick Institute, London, UK; h Structural Biology Science Technology Platform, The Francis Crick Institute, London, UK; i Cancer Research UK Beatson Institute, Glasgow, UK; j Munich Cluster of Systems Neurology, Ludwig-Maximilians-Universität München, München, Germany; k Autophagy, Inflammation and Metabolism Center of Biochemical Research Excellence, Albuquerque, NM and Department of Molecular Genetics and Microbiology, University of New Mexico Health Sciences Center, Albuquerque, NM; l Department of Biomolecular Sciences, The Weizmann Institute of Science, Rehovot, Israel; m Institute of Biochemistry II, Medical Faculty, Goethe-University, Frankfurt am Main, and Buchmann Institute for Molecular Life Sciences, Frankfurt am Main, Germany; n Autophagy Research Group, Department of Medical Biology, University of Tromsø - The Arctic University of Norway, Tromsø, Norway

**Keywords:** Autophagy, Atg8, AIM, LIR, LDS, UDS, protein-protein interaction, phosphorylation

## Abstract

The Atg8 family of ubiquitin-like proteins play pivotal roles in autophagy and other processes involving vesicle fusion and transport where the lysosome/vacuole is the end station. Nuclear roles of Atg8 proteins are also emerging. Here, we review the structural and functional features of Atg8 family proteins and their protein-protein interaction modes in model organisms such as yeast, *Arabidopsis, C. elegans* and *Drosophila* to humans. Although varying in number of homologs, from one in yeast to seven in humans, and more than ten in some plants, there is a strong evolutionary conservation of structural features and interaction modes. The most prominent interaction mode is between the LC3 interacting region (LIR), also called Atg8 interacting motif (AIM), binding to the LIR docking site (LDS) in Atg8 homologs. There are variants of these motifs like “half-LIRs” and helical LIRs. We discuss details of the binding modes and how selectivity is achieved as well as the role of multivalent LIR-LDS interactions in selective autophagy. A number of LIR-LDS interactions are known to be regulated by phosphorylation. New methods to predict LIR motifs in proteins have emerged that will aid in discovery and analyses. There are also other interaction surfaces than the LDS becoming known where we presently lack detailed structural information, like the N-terminal arm region and the UIM-docking site (UDS). More interaction modes are likely to be discovered in future studies.

## Introduction

The identification of the small ubiquitin-like protein Atg8 as a core autophagy protein came from early studies of essential autophagy-related (ATG) proteins in the budding yeast *Saccharomyces cerevisiae* [1]. Autophagosome formation is induced by the activation of two kinase complexes, i. e. Atg1/ULK (unc-51 like autophagy activation kinase 1) and PIK3C3 (phosphatidylinositol 3-kinase catalytic subunit type-3), and it also depends on Atg9-containing vesicles [2]. Their activation results in a local formation of phosphatidylinositol-3-phosphate (PI3P) at a pre-autophagosomal structure or phagophore-assembly site (PAS) close to ER, and a double membrane structure named phagophore, or isolation membrane, is formed. A complex containing Atg18/WIPI and Atg2 is recruited to mediate phagophore expansion directly by the lipid transferase activity of Atg2 [3], and to aid Atg9 vesicles in the delivery of phospholipids from the ER to the phagophore. ATG9A promotes phagophore expansion by acting as a lipid scramblase [4,5]. Autophagosome formation also relies on two different conjugation systems. The ubiquitin like protein Atg12 is conjugated to Atg5, and the Atg12-Atg5 forms a complex with Atg16 that is recruited to PAS. This complex acts as an E3 ligase for the conjugation of Atg8 to the phagophore membrane. Atg7 is the E1 in both conjugation reactions, while Atg10 and Atg3 works as E2 for Atg12 and Atg8, respectively [6]. On the phagophore membrane, Atg8 acts as a scaffold for the recruitment of core autophagy proteins to the phagophore and this facilitates phagophore expansion. A closure of the expanded phagophore results in autophagosome formation, and cytoplasmic material engulfed by the autophagosome is degraded upon fusion of the autophagosome with a lysosome [2,7].

The role of Atg8 in macroautophagy is evolutionary conserved, but the number of Atg8 orthologues vary between species from a single family member in yeast to more than 10 variants in higher plants. In metazoans, the family members are divided into two subgroups, i. e. microtubule -associated protein 1 light chain 3 (LC3) and γ-amino-butyric acid receptor-associated protein (GABARAP). The GABARAP subgroup is evolutionary conserved, while the LC3 subgroup is metazoan specific [6,8]. Atg8 orthologues are in all species produced as a pro-form lacking a C-terminal glycine residue needed for the conjugation reaction. The pro-form (pro-Atg8) is activated by proteolytic cleavage by cysteine proteases of the Atg4 family. The resulting I form with a C-terminal glycine residue exposed can then be lipidated to phosphatidylethanolamine (PE) on the outer membrane surface of the phagophore membrane, and this creates the II form that is an adaptor for the recruitment of other proteins involved in phagophore expansion, including other core autophagy proteins. The presence of Atg8 proteins is believed to be most important at the rim of the phagophore since this is where phagophore growth occurs. The closure of the expanded phagophore correlates with a partial de-lipidation of the Atg8 coat by Atg4 family proteases [6]. However, some Atg8 proteins remain on the completed autophagosome to facilitate recruitment of proteins involved in transport or fusion of the autophagosome with a lysosome. Atg8 proteins are also lipidated to the inner membrane surface of the phagophore where they act as adaptors for the binding of selective autophagy receptors (SARs)[9]. In selective autophagy, a cargo selected for degradation by autophagy must first be identified and bound by a SAR. Different types of SARs have been identified that associate with different types of cargos, but a common feature is the direct binding of the SAR to Atg8 proteins attached to the inner membrane surface of the growing phagophore. This way, the SAR is responsible for the tight docking of the selected cargo to the inner phagophore membrane [9].

Most studies on Atg8 proteins have focused on their role(s) in macroautophagy, but several recent studies have shown that Atg8 proteins can be lipidated to single membranes to participate in non-conventional autophagy pathways, secretory autophagy, or endocytic processes like LC3-associated phagocytosis (LAP), entosis, micropinocytosis or endocytosis. The term “atg8ylation” has been coined to emphasize the analogy to ubiquitin since proteins can be atg8ylated by being covalently bound to the C-terminal glycine of Atg8 proteins [10], and ubiquitin can be conjugated to PE [11]. Atg8ylation is used as a response to membrane stress and membrane remodeling activities both in autophagy and in other processes involving atg8ylation to single membranes including lysosomal damage responses [10,12]. There is also increasing evidence for that unlipidated Atg8 proteins may have functional roles, including reported nuclear roles displayed by nuclear pools of these proteins [13-16]. Of note, a mammalian Atg8 protein, GABARAPL2 (also known as GATE-16), was identified first as a SNARE-interacting protein, before its engagement in autophagy became appreciated. GABARAPL2, in association with the Golgi SNARE GOSR1 (GOS-28) and the SNARE complex-unfolding protein NSF [17-20], controls intra-Golgi transport , protein trafficking to the plasma membrane, and post-mitotic Golgi reassembly. Expanding upon this early work, recent studies show direct interactions of all LC3 and GABARAP proteins with an assortment of other SNARE proteins, including SNAREs that regulate autophagosome and lysosome biogenesis [21,22].

The aim of this review is to summarize the knowledge we have on binding motifs and interaction surfaces utilized by Atg8 orthologues and interacting proteins. Most characterized interactions depend on a small sequence motif in the interacting protein that was initially named LC3 interacting region (LIR) following its discovery in human p62/SQSTM1 [23]. Later studies revealed that this motif is evolutionary conserved, and often referred to as Atg8 interacting motif (AIM) in yeast, fungi and plants [24]. The LIR/AIM motif docks into a so-called LIR docking site (LDS) that is conserved in all Atg8 orthologues. An overwhelming majority of known interactions with Atg8 proteins involve LIR motifs [9]. The LIR-LDS interaction will therefore be discussed in detail in this review, but we will also discuss other known interactions relevant for the function of Atg8 proteins.

## Yeast Atg8

**Figure 1.**
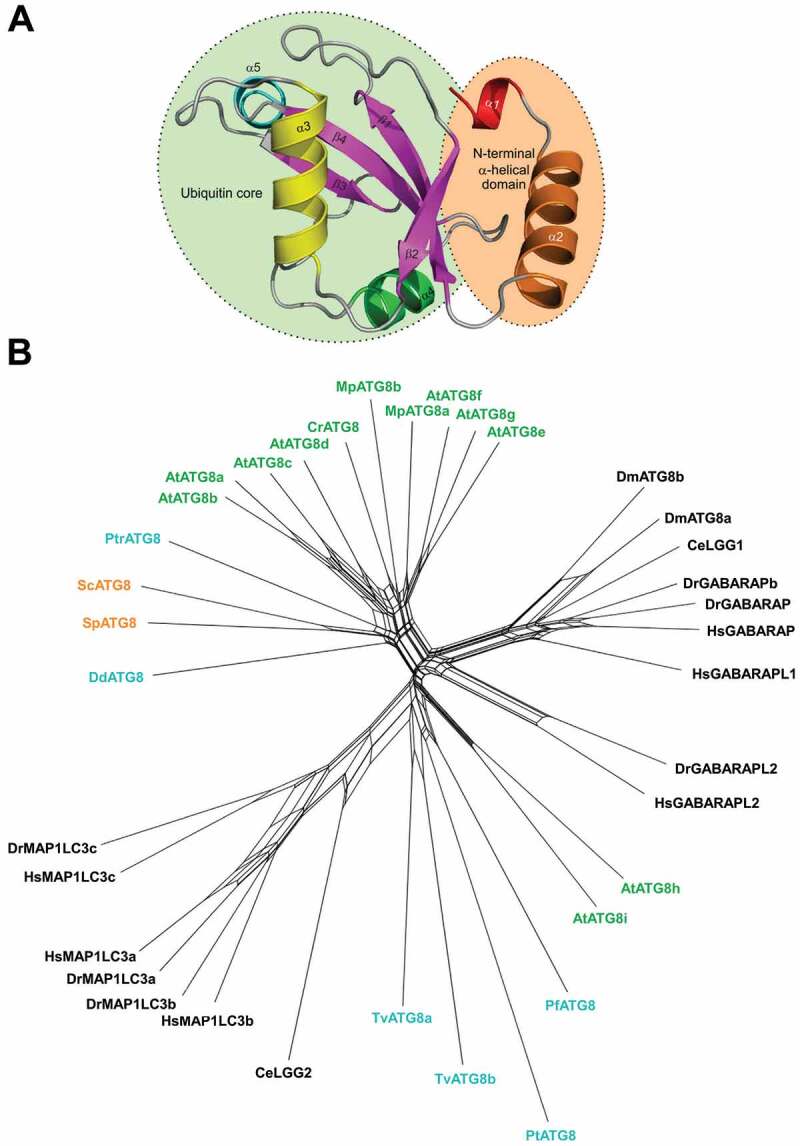
*Structure and diversity of Atg8 family proteins*. (**A**) Structural organization of the Atg8/LC3/GABARAP proteins. (**B**) Neighbor-net analysis of 36 ATG8 nucleotide sequences from 14 eukaryotic species. The network was calculated with Splitstree4 applying the general time reversible (GTR) distance matrix using a conserved 332 nt position calculated with Gblocks from an original alignment of 666 nt positions in MUSCLE. Species labels are colored according to a major grouping into plant (green), fungal (orange), animal (black) or protozoan (blue) lineages. (Hs, *Homo sapiens*; Sc, *Saccharomyces cerevisiae*; D.m, *Drosophila melanogaster*; C.e, *Caenorhabditis elegans*; Dr, *Danio rerio*; At, *Arabidopsis thaliana*, Mp, *Marchantia polymorpha*; Cr, *Chlamydomonas reinhardtii*; Pt, *Paramecium tetraurelia*; Dd, *Dictyostelium discoideum*; Pf, *Plasmodium falciparum*; Pt, *Phaeodactylum. tricornutum*; Tv, *Trichomonas vaginalis*)

As mentioned above, Atg8 was first identified in *S. cerevisiae* as a protein required for autophagy and the cytoplasm-to-vacuole targeting (Cvt) pathway [25]. Atg8 has in its core a ubiquitin-like fold with an N-terminal extension (**Figure 1A**). In this organism, Atg8 is synthesized as a proform with a single arginine (Arg117) at the C terminus, which is immediately removed by the cysteine proteinase Atg4 [26]. The newly exposed C-terminal glycine (Gly116) is activated by the E1 enzyme Atg7 with consumption of ATP and forms a thioester bond with the cysteine residue (Cys507) of Atg7. Then, the Gly116 of Atg8 is transferred to the cysteine residue (Cys234) of the E2 enzyme Atg3 and finally forms an amide bond with the amino group in the hydrophilic head of PE [27]. In this final step of Atg8 lipidation, the Atg12-Atg5-Atg16 complex serves as an E3 enzyme, which stimulates the transfer of Atg8 from Atg3 to PE and thereby confines the reaction to the PAS and phagophore where the complex localizes [28-30]. Atg8 conjugation to PE is reversible; the amide bond in Atg8-PE is cleaved by Atg4, releasing Atg8 from membranes [26]. This deconjugation reaction maintains a reservoir of unlipidated Atg8 and promotes autophagosome formation [31-34].

The molecular functions of Atg8, especially its lipidated form, have been studied using in vitro reconstitution system [35-40]. Initial reconstitution experiments were performed using Atg7, Atg3, processed form of Atg8, small unilamellar vesicles (SUVs) containing high content of PE, and MgATP, which were shown to be sufficient for Atg8-PE formation *in vitro* [41]. Later, addition of the Atg12-Atg5 conjugate to this reconstitution system was shown to dramatically accelerate Atg8-PE formation, even when using SUVs with physiological content of PE, establishing that the Atg12-Atg5 conjugate is the E3 enzyme for the Atg8 system [28]. In the reconstitution system, progression of lipidation reaction caused multimerization of Atg8, which induced tethering and hemi-fusion of SUVs to each other. Moreover, mutations inhibiting these Atg8 activities partially or completely resulted in generation of smaller or no autophagosomes in yeast, respectively. These observations suggested that Atg8-PE possesses membrane tethering and hemi-fusion activities proposed to represent its function in autophagosomal membrane expansion [39]. When giant unilamellar vesicles (GUVs) were used instead of SUVs, the Atg12-Atg5 conjugate required Atg16 to function as the E3 enzyme for Atg8-PE formation as observed *in vivo*, confirming that Atg16 is also a critical component of the E3 enzyme of the Atg8 system [40]. Monitoring the time-course of GUV targeting revealed that Atg8-PE on GUVs has the activity to recruit the Atg12-Atg5-Atg16 complex to GUVs, which further promotes Atg8-PE formation as a positive feedback loop [35]. In addition to the monitoring of protein localization, utilization of GUVs enabled the monitoring of membrane morphology upon Atg8-PE formation. Formation of Atg8-PE caused membrane tubulation in addition to tethering of spherical GUVs [36]. When non-spherical, prolate GUVs were used, Atg8-PE caused dramatic shape change of prolate GUVs into a sphere without-bud. This shape change was dependent on the membrane-perturbation activity of Atg8-PE to increase area difference between outer and inner leaflets of GUVs. The mutations that impaired this *in vitro* activity resulted in smaller and fewer autophagosomes in yeast, indicating its importance in autophagosome formation [37].

Besides its lipidation-dependent roles in autophagy, Atg8 is known to play lipidation-independent roles that are not related to autophagy. In yeasts such as *S. cerevisiae, Schizosaccharomyces pombe*, and *Komagataella phaffii*, Atg8 plays a critical role in maintaining the vacuole morphology independently of conjugating enzymes [42-44]. In *S. cerevisiae* and *S. pombe*, Atg8 was recruited to the vacuole via interaction with the vacuolar membrane protein Hfl1 and they collaboratively contributed to the fragmented vacuolar morphology under stress such as dithiothreitol treatment [42]. This non-autophagic role was shown to require the membrane perturbation activity of Atg8 [37]. In *Drosophila melanogaster* lipidated Atg8a is required for autophagy, while its non-lipidated form is essential for developmentally programmed larval midgut elimination and viability. Additionally, high expression of non-lipidated Atg8b in the male germline is required for fertility (see also the section on Atg8s in *Drosophila melanogaster*)[45].

In addition to autophagosome formation, Atg8 also plays an important role in cargo sequestration into autophagosomes during selective autophagy in yeasts as well as other organisms. In *S. cerevisiae*, Atg8 binds to the AIMs/LIRs in the autophagy receptors Atg19, Atg34, Atg32, Atg36, Atg39, Atg40, and Cue5 [46-55]. While Atg19 recognizes vacuolar enzymes (Cvt cargos), α-mannosidase and Ty1 virus-like particles, Atg34 specifically interacts with α-mannosidase. Atg32, Atg36, Atg39 and Atg40 localize to mitochondria, peroxisomes, the nucleus , the endoplasmic reticulum (ER), and initiate autophagic degradation of these organelles, respectively. Cue5 mediates sequestration of polyQ proteins and inactive proteasomes[46-55]. In selective autophagy of peroxisomes (pexophagy) in *K. phaffii* and *Candida boidinii*, Atg8 cooperates with the pexophagy receptor Atg30 [56,57]. In *S. pombe*, selective sequestration of mitochondria and the ER into autophagosomes is mediated by the interaction of Atg8 with Atg43 and Epr1, respectively [58,59]. *S. cerevisiae* Atg8 also directly (not via autophagy receptors) binds to selective autophagy cargos such as the endocytic protein Ede1, fatty acid synthase, and nuclear pore complexes [60-63].

In *S. cerevisiae*, the expression of Atg8 was shown to be transcriptionally upregulated upon nitrogen starvation or the inactivation of mechanistic target of rapamycin complex 1 (mTORC1) [64,65]. The Rpd3-Sin3-Ume6 histone deacetylase complex binds to the promoter region of the *ATG8* gene to repress its transcription under nutrient rich conditions. A similar mechanism is likely to work in mammalian cells [64]. Increased levels of Atg8 result in an increase in the size of autophagosomes [64,66].

## Plant Atg8s

Like the yeast Atg8 orthologue, plant Atg8s also contain a ubiquitin fold, the N-terminal extension, and the C-terminal glycine that serves as a recognition site for Atg4 protease [67]. However, unlike yeast, plants have multiple Atg8 homologs (**Figure 1B** and**Figure 2**), ranging from 2 homologs in the emerging model system *Marchantia polymorpha* to 9 homologs in *Arabidopsis thaliana* and up to 22 homologs in the *Arabidopsis* relative plant *Capsella rubella* and *Brassica napus* [68,69]. These homologs form two well-supported evolutionary clades as seen for the *Arabidopsis* Atg8s in **Figure 1B**, where Clade I is closely related to fungal Atg8s, whereas Clade II groups, represented by AtATG8i and -h, cluster together with metazoan Atg8 orthologues [68]. Surprisingly, some of the homologs in Clade II lack any residues after the C-terminal glycine (**Figure 2**), but they still associate with Atg4, consistent with recent finding suggesting that Atg4 can promote autophagosome formation, independent of its protease activity [70-72]. Another interesting feature of plant Atg8 phylogenetic tree is the formation of family specific Atg8 subclades. For example, *Brassicaceae* family that contains *Arabidopsis* forms 9 monophyletic subclades, whereas *Poaceae* family that contains wheat forms 4 subclades. Each subclade contains fixed polymorphisms, suggesting they maybe functionally diversified [68].

**Figure 2.**
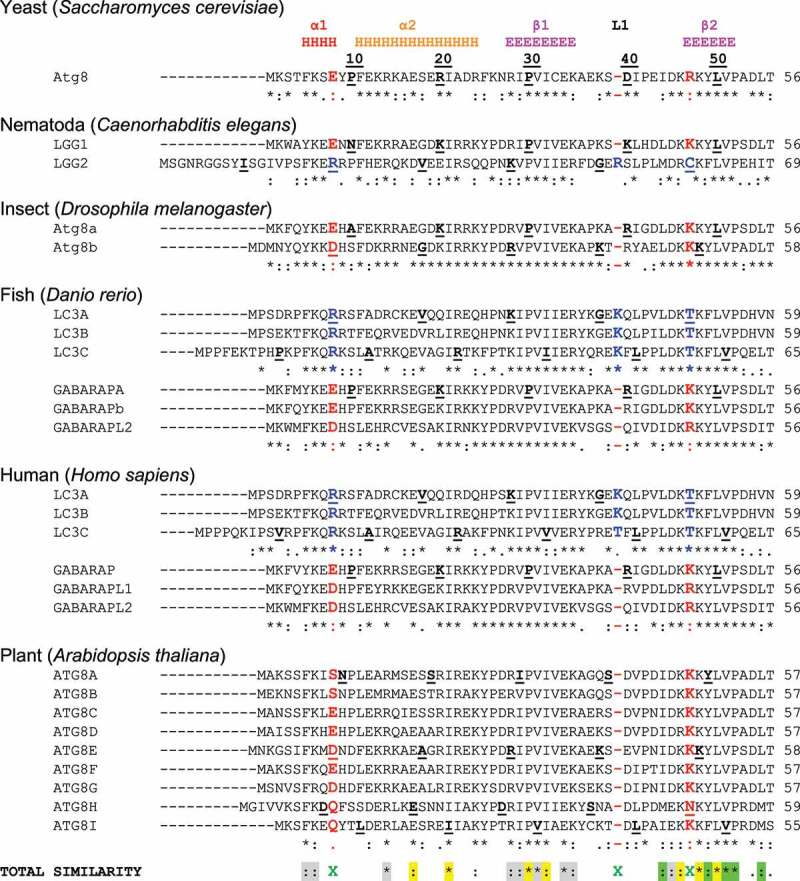
*Sequence alignment of Atg8/LC3/GABARAP proteins*. Sequence alignment of the Atg8-family members from 6 model species – yeast (*Saccharomyces cerevisiae*), nematoda (*Caenorhabditis elegans*), insects (*Drosophila melanogaster*), bony fish (*Danio rerio*), human (*Homo sapiens*) and plant (*Arabidopsis thaliana*). Secondary structure elements from the human LC3B (PDB ID 2ZJD) are shown at the top (H – α-helices, E – β-strands, L – long loops; rainbow color-code for α-helices – red, orange, yellow, green and cyan, all β-strands are in magenta). Every tenth residue in each sequence is marked bold/underlined, the catalytic Gly is marked green. The identity scores (* for identical residues,: for very similar residues, for analogous residues, space for residues without any similarity, - for gaps) are presented below each group of the Atg8/LC3/GABARAP. For the yeast Atg8 proteins, annotated UniProt entries for 11 yeast species Atg8 sequences were aligned to generate the identity score. The residues (or their absence) separating GABARAP/Atg8 and LC3 protein subtypes are marked red and blue, respectively. The consensus string for all 38 proteins aligned is presented at the bottom of alignment (named TOTAL SIMILARITY). The residues showed conservation are grouped within the following classes: residues participating in the protein folding (grey); residues forming HP1 (yellow); residues forming HP2 (light green); and residues forming UDS (cyan). The key residues indicating LC3 and Atg8/GABARAP subtypes difference are marked by green X.

**Figure 2.**
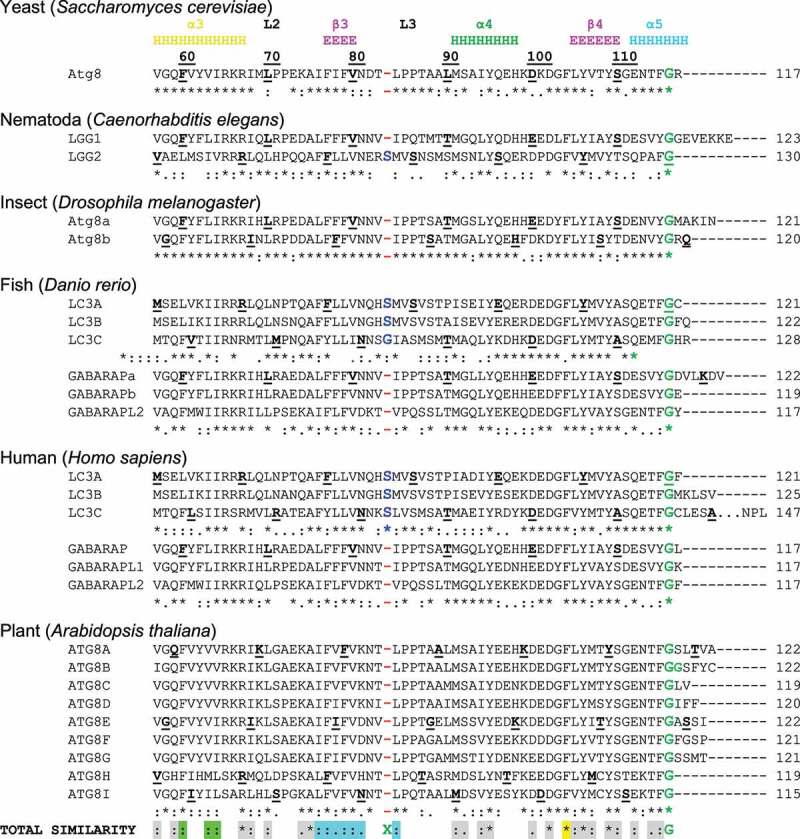
(Continued).

Similar to the metazoan Atg8 homologs, recent studies have shown that plant Atg8s are also functionally specialized [72,73]. Domain swap analysis and interactome studies performed using potato Atg8s have shown that the N terminal β-strand that forms a part of the hydrophobic pocket 1 (HP1) at the LIR docking site (see “LIR-LDS interaction” below) underpins specialization towards a pathogen effector protein and plant proteins. Further genetic studies are necessary to show if Clade I or Clade II Atg8 homologs have specific functions in macroautophagy or non-canonical forms of autophagy.

## Atg8s in *Caenorhabditis elegans*

*C. elegans* contains two Atg8 homologs, LGG-1 and LGG-2, that have differential functions in autophagy. They are also structurally different. LGG-1 is similar to the GABARAP subfamily and LGG-2 more similar to the LC3 subfamily (**Figure 1B**) [74]. LGG-1 is synthesized as a 123-amino acid precursor, whose C-terminal seven amino acids are cleaved by ATG-4 to expose the glycine for PE conjugation. In embryonic extracts, the unlipidated processed form (LGG-1-I) and the lipidated processed form (LGG-1-II) are detected, while the LGG-1 precursor is absent [75]. The LGG-1 precursor is processed by the two Atg4 homologs in *C. elegans*, ATG-4.1 and ATG-4.2. ATG-4.1 cleaves LGG-1 precursors about 100-fold more efficiently than ATG-4.2 in *in vitro* cleavage assays [75]. Compared to wild-type animals, LGG-1 is properly processed and lipidated in *atg-4.2* mutants, while in *atg-4.1* mutants, LGG-1 precursors accumulate dramatically, lipidated LGG-1-II is present at a similar level, but unlipidated LGG-1-I is absent [75]. In LGG-2, the glycine for conjugation is directly exposed (**Figure 2**). However, the unlipidated form of LGG-2 is predominant in embryonic extracts [74].

LGG-1 and LGG-2 form spatiotemporally dynamic punctate structures during *C. elegans* embryogenesis. Loss of function of autophagy genes acting at different steps of the autophagy pathway results in characteristic levels of lipidated LGG-1 and formation of LGG-1 puncta. In mutants of the conjugation systems, including *atg-3, atg-7, atg-5* and the *atg-4.1; atg-4.2* double mutant, the lipidated forms of LGG-1 and LGG-2 are not detected and formation of LGG-1 and LGG-2 puncta is abolished [75,76]. ATG-16 is not required for LGG-1 lipidation, but is essential for formation of LGG-1 puncta in *C. elegans* [77]. In UNC-51/EPG-1/EPG-9 Atg1 complex mutant embryos, LGG-1-I accumulates and LGG-1 puncta are largely absent except in a few cells which contain large aggregates [78]. In *epg-8* (encoding *C. elegans* Atg14 homolog) or *bec-1* mutants, levels of LGG-1-I and LGG-1-II are elevated but LGG-1 puncta are weaker [79]. *atg-9* mutant embryos have larger but fewer LGG-1 puncta than wild-type embryos [80]. In loss-of-function mutants of genes downstream of autophagosome initiation, including *epg-3, epg-4, epg-6, atg-2* and *epg-5*, lipidated LGG-1 and LGG-1 puncta accumulate [76,81]. *lgg-2* mutants exhibit a wild-type pattern of LGG-1 punctum formation, while loss of *lgg-1* dramatically increases the number of LGG-2 puncta [74]. Modulating the lipidation of one Atg8 homolog by another provides a novel mechanism for regulating their differential functions in the pathway.

LGG-1 and LGG-2 differentially bind to autophagy cargos such as SQST-1, SEPA-1 and AIN-1 [74]. The scaffold proteins EPG-7 and EPG-2, which mediate degradation of SQST-1 and PGL granules, respectively, bind strongly to LGG-1, but show no or weak interaction to LGG-2 [74]. The Atg1 complex components UNC-51 and EPG-1 preferentially interact with LGG-1, while LGG-2, but not LGG-1, directly interacts with the LGG-3/ATG-5/ATG-16 complex [74]. EPG-5, which acts as a tethering factor to promote fusion of autophagosomes with late endosomes/lysosomes, directly interacts with LGG-1 [82]. Their differential interactions with autophagy proteins may contribute to hierarchical recruitment of Atg proteins in the autophagy pathway.

## Atg8s in *Drosophila melanogaster*

Contrary to the significantly more extended and diverse family of ubiquitin-like ATG8 proteins in mammals, the *Drosophila* Atg8 group is represented by two members: Atg8a and Atg8b. Both are structurally more similar to GABARAP than LC3, they are upregulated in response to autophagy induction, colocalize to autophagosomes and share at least some degree of redundancy as seen from loss-of-function alleles that present milder autophagy-mutant phenotypes than otherwise expected for loss of this central Atg protein [83-86]. Despite these similarities, the Atg8a isoform shows the more ubiquitous expression, while Atg8b is mostly relegated to the adult male testis [45]. As such, the bulk of assays that monitor autophagy in *Drosophila*, use Atg8a as the reference marker [87-89]. In terms of ontology, Atg8b most probably originated from a retrotransposition event of Atg8a during the emergence of fruit flies, as it lacks the intron regions of the latter [90]. The tissue-specific enrichment of Atg8b in the testis supports this theory, as it is in line with how the process of late spermatogenesis and inactivation of the X chromosome, that occurs in both *Drosophila* and mammals, can result in the generation of autosomal retrogenes from X-linked genes (such as Atg8a) with male germline-specific localization [90]. Closely related to the process of spermatogenesis, in the fruit fly male testis, Atg8b has mostly foregone its critical requirement in autophagy for a non-autophagic, lipidation-independent role, as it is essential for viable sperm production and regulation of male fertility [45]. Of note here, in this setting, overexpression of Atg8a in Atg8b-null mutants was able to restore male fertility, suggesting that both fly Atg8 proteins can mediate their non-autophagic effects with regards to spermatogenesis [45].

An added layer of complexity in the mammalian system is that the LC3 and GABAPAP subfamilies have further evolved to perform distinct functions in autophagy and by extension their members are encountered at different stages of the process [91]. In contrast Atg8a, is found throughout all stages of autophagy in *Drosophila*, which can at times simplify the monitoring of autophagy in this model organism compared to others.

## Human, mammalian and other metazoan Atg8s

Humans express seven ATG8 orthologues of which three are in the GABARAP subfamily. This subfamily is again separated into two subgroups, i. e. one consisting of GABARAP and GABARAPL1 and the other by GABARAPL2. The LC3 subfamily is similarly divided into two subgroups, i. e. one consisting of LC3A (two splice-isoforms with differing N-terminals), LC3B and LC3B2, and one consisting of LC3C (**Figure 1B**). Intriguingly, rodents lack LC3C [92]. All human ATG8s are formed as precursors that must be cleaved by ATG4 to expose the glycine for PE lipidation. Four different ATG4 cysteine proteases (A-D) exist in human cells, and they differ in specificity related to the processing or delipidation of human ATG8 orthologues. The binding between ATG4 and ATG8 orthologues is interesting since it involves two different interactions. One involves the catalytic domain in ATG4 [93], and the other a LIR motif at the C-terminus of ATG4 [94]. A number of other human core autophagy proteins also have a LIR motif. Unlike the motif in ATG4B which has a broad binding specificity, the motifs in other core autophagy proteins have a binding preference for GABARAP and GABARAPL1 [95-100], suggesting an important role for this subgroup of ATG8s in autophagosome formation. This is also the subgroup that has the highest similarity to protist and yeast Atg8s. Hence, it comes as no surprise that this subgroup is important for autophagosome formation. CRISPR/CAS9 knockout studies show that autophagosomes are formed even without mammalian Atg8s, although the autophagy is very inefficient, and LC3/GABARAP proteins are needed for efficient autophagosome-lysosome fusion [101]. Rescue experiments underscored the importance of the GABARAP subfamily for selective autophagy and starvation induced autophagy [101,102]. Knock down studies suggested the LC3 subfamily to be dispensable, while the GABARAP subfamily is required for bulk autophagy [103]. The LC3 subfamily is suggested to be more important as an adaptor for the docking of cargos to the phagophore membrane in selective autophagy, and it may also be involved in the transport of endosomes and autophagosomes whereas GABARAPs are involved in fusion between autophagosomes and lysosomes. However, the interplay between the different human ATG8s is complex and remains poorly understood, and there may exist functional redundancy between them [9].

During evolution, the four unique Atg8 subgroups in mammals appear to be derived from an early metazoan lineage split of Atg8 orthologues. For example, a primitive animal like Hydra, a freshwater *Hydrozoa* of the *Cnidarium* phylum, has four Atg8 orthologues that are homologous with each of the subgroups found in mammals, i. e. GABARAP, GABARAPL2 , LC3A/B and LC3C [104]. A similar conservation of Atg8 subgroups is not seen throughout the metazoans. Due to divergent evolution, two important model organisms, i. e. the fruit fly *Drosophila* and the nematode *C. elegans* have retained only two Atg8 homologs representing only one and two of the subgroups, respectively [45].

## The LIR – LDS interaction

The LIR/AIM motif binds to LDS and is universally used for Atg8 protein interactions throughout eukaryotic evolution. To describe the LIR/AIM-LDS interaction, we will initially focus on mammals and yeast since the vast majority of identified LIRs are from mammals and most structures are on mammalian and yeast LIR/AIM-LDS interactions.

**Figure 3.**
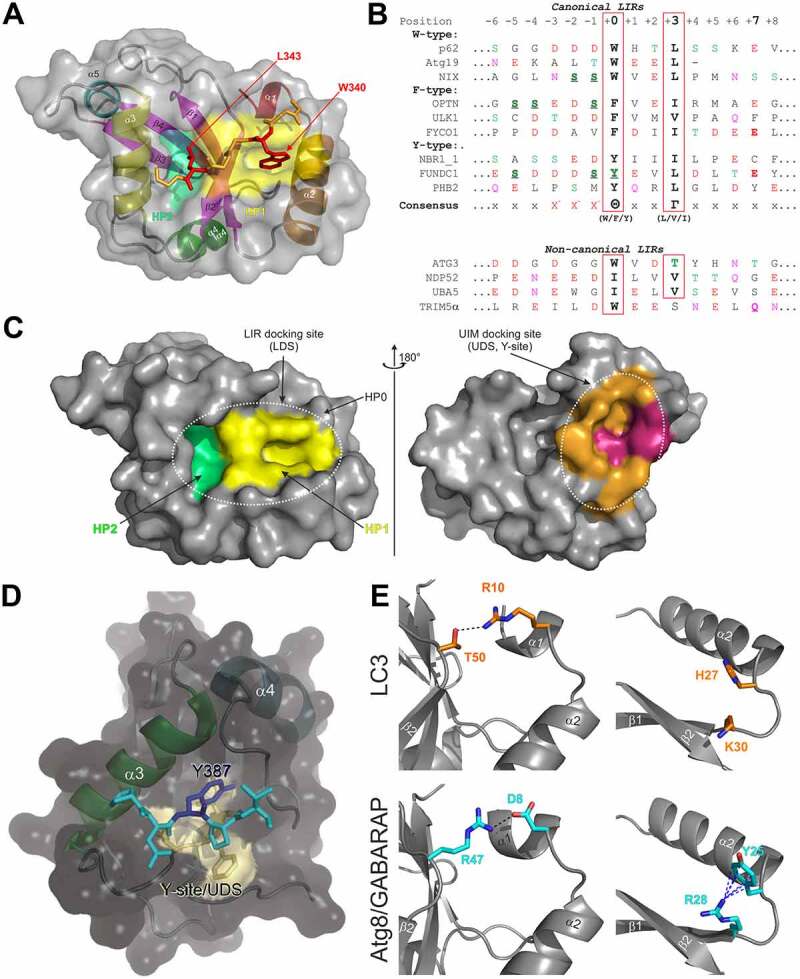
*Atg8/LC3/GABARAP interactions with their partners*. (**A**). Structure of p62/ SQSTM1-LIR:LC3B complex (PDB ID 2ZJD). Human LC3B is shown as a semi-transparent surface with the structural elements (α-helices and β-strands) visible. Murine p62/SQSTM1-LIR is shown as a main chain (orange) with sidechains of core LIR residues (W340 and L343, red) as sticks. Two hydrophobic pockets of LC3B, accommodating W340 and L343 sidechains, are shown on LC3B surface (HP1 – yellow, HP2 – light green). (**B**) Alignment of canonical (top) and non-canonical (bottom) LIR motifs with positions of residues indicated on top (from -6 to +8). Negatively charged residues (red), polar residues (magenta) and phosphorylatable residues (green) are indicated over the LIR sequences. The phosphorylatable residues confirmed to be phosphorylated are marked bold and underlined (see Table 1 for details). Residues at positions 0 and +3 within core LIR sequences whose sidechains are accommodated by HP1 and HP2 are boxed. Glutamate residues at position +7, forming additional intermolecular hydrogen bond to Arg/Lys at α-helix α3 in LC3/GABARAP proteins, are marked bold. Note that the enumeration in this work is according to N. Noda with the aromatic residue Θ as position 0, whereas this position is often numbered as +1 in many papers. (**C**) Interaction sites on Atg8/LC3/GABARAP surface. Left plot: surface representation of LC3B structure (the same orientation as in (A)), showing the main interacting sites - HP1 (yellow) and HP2 (light green), which form the LC3 docking site (LDS). Position of additional interacting site, HP0, is indicated by arrows. The alternative interacting area, the UIM docking site (UDS, including Y-site), is located on the opposite side of the LC3B molecule (right plot). The most relevant residues are colored dark red, additional hydrophobic residues around it are colored orange. (**D**) Structure of Hfl1 bound to the Y-site/UDS of Atg8 (PDB ID 6AAG). (**E**) Structural differences between LC3 and GABARAP proteins. (Right) Intramolecular contacts within LC3B (top) and GABARAP (bottom) proteins. Involved residues are presented as sticks, intramolecular hydrogen bonds between these residues are shown as black dashed lines. (Left) Orientation of H27 and K30 sidechains in LC3B (top) and corresponding Y25 and R28 sidechains in GABARAPs (bottom). Cation-π interactions (the non-covalent electrostatic interaction between an electron-rich face of aromatic rings and adjacent cations), stabilizing the specific orientation of Y25/R28 sidechains in GABARAPs are shown as blue dashed lines.

The LIR in mammals and AIM in yeast were described in pioneering biochemical [23] and structural [105,106] works, as short polypeptide sequences containing ~10 residues. Early structural studies revealed that the core LIR/AIM (LIR hereafter) sequence contains a W-X-X-L motif (where X is any residue). The LIR polypeptide of p62/SQSTM1, for instance, adopts a β-stranded conformation, forming an intermolecular parallel β‐sheet with the β-strand β2 of LC3B, while the sidechains of W and L residues occupy the two hydrophobic pockets - HP1 (also known as W-site) and HP2 (L-site) - on LC3B surface, stabilizing the complex (**Figure 3A**). Extensive studies in past years provided a more general core consensus, which can be described as Θ_0_-X_1_-X_2_-Γ_3_(or positions 0, +1, +2 and +3), where Θ is an aromatic (W/F/Y) and Γ is a hydrophobic (L/I/V) residue (**Figure 3B**). Investigations of the residues which could occupy the Θ and Γ positions (either by analyzing the sequences of the hitherto known canonical LIR motifs [9] or by mutational 2D peptide arrays [95,96,100,107] revealed a very high conservation of the three aromatic residues in Θ. As expected from the hydrophobicity profile of HP1, a much higher abundance of solely non-polar Trp and Phe was observed in the native canonical LIRs. In contrast, partially polar Tyr residues are found in only a minority of canonical LIR motifs. Trp is the most energetically favorable residue for the Θ position. Mutation of the Tyr732 to a Trp increases the NBR1 LIR affinity to GABARAPL1 8-fold, while the Y732F mutant showed the same affinity [108]. The Phe-containing OPTN LIR shows an 8-fold increase in affinity to LC3B when Phe178 is substituted to a Trp [109]. Of note, the lower affinity of Tyr- and Phe-containing canonical LIR motifs in both aforementioned cases might be associated with the ability of NBR1 and OPTN to regulate autophagic functions [108,109]. The Γ position is a bit less conserved and tolerates large hydrophobic residues, including canonical L/V/I and aromatic residues, except His. Apparently, smaller hydrophobic residues, such as Ala, Pro or Met, do not have enough volume to fill the HP2, while aromatic residues can be too big to be docked (but still could be present at the Γ position (+3)) [110-112]. There is a Met residue in this position in a very few LIRs [113]. Also, for some LIRs, e.g. PCM1, ULK1, or Atg14, aromatic residues are likely able to dock to HP2 as Phe in that position results in good binding in peptide arrays [96,100].

Another functionally important feature of LIR sequences is the presence of negatively charged (Glu/Asp) or phosphorylatable (Ser/Thr) residues prior to the core motif at position -1 or at positions -2 or -3 (**Figure 3B**). Mutational analyses have shown that their presence strongly enhances the affinity of LIR interactions with Atg8/LC3/GABARAP [9,23].

## Structural features of the LIR-LDS interaction

The main structural difference between Atg8/LC3/GABARAP proteins and other UBLs, which also determines the specific role of Atg8/LC3/GABARAP in autophagy, is the presence of two additional α-helices located N-terminally to the ubiquitin core (**Figure 1A**). This was revealed with the determination of the first Atg8 structure, the one of GABARAPL2/GATE-16 [114]. This N-terminal α-helical subdomain significantly varies in the amino-acid content among the different members of the Atg8/LC3/GABARAP family, and structural studies indicate that it displays a dynamic behavior, participating in a conformational exchange [115-117]. Consequently, this structural adaptation of Atg8/LC3/GABARAP is reflected in a set of new functions not observed for other UBLs. For instance, the N-terminal α-helices are essential for tubulin binding and oligomerization [115], strengthening tethering of lipid bilayers upon autophagosome maturation [39,118], and recognition of mitochondrial phospholipids [119].

Despite their flexibility, the N-terminal α-helices are specifically aligned to the ubiquitin-like core, forming the deep HP1, (also termed W-site) together with residues of the β-strand β2 (**Figure 3A**). This pocket binds preferentially indole-based substances, albeit with low affinity [120], and usually accommodates large sidechains of non-polar aromatic residues within the LIRs. HP1 is formed by residues D19, I23, P32, I34, K51, L53, and F108 in LC3B [106]. HP2 (L-site), is built by hydrophobic residues of the central α-helix α3 and β-strand β2 (F52, V54, P55, L63, I66, and I67). These two pockets form the LDS and mediate a vast majority of known-to-date interactions between SARs, adaptors and scaffolding proteins with Atg8/LC3/GABARAPs [9].

Besides the LDS, Atg8/LC3/GABARAP possess an interaction site at the opposite molecular surface. This site, called the UIM-docking site (UDS) (**Figure 3C**) is similar to the well-known hydrophobic patch (L8-I44-V70) of ubiquitin [121], and contains a number of surface-exposed hydrophobic residues around F79 and L81 in LC3A and LC3B corresponding to L76 and F78 in GABARAPs. The UDS is used by components of the UPS machinery (such as RPN10) and during intracellular trafficking (such as Ataxin-3 and EPS15) [122]. The unlipidated form of yeast Atg8 also utilizes the UDS (named Y-site since it accommodates Tyr) in addition to LDS for binding the non-canonical LIR of Hfl1, which collaboratively regulates vacuolar morphology under stress as mentioned above (**Figure 3D**)[42]. Interactions involving the UDS are further discussed in separate sections below.

The structural differences within the human Atg8-family proteins are rather small - the backbone of the core regions of human LC3/GABARAP proteins can be overlaid with a RMSD of 1.2 Å. Nevertheless, structural differences exist and reflect mostly differences in Atg8/LC3/GABARAP sequences (**Figure 2**), therefore, it is important to further analyze these sequence deviations to define and understand the structural differences, which may confer functional differences as well. There are substantial differences in the sequences not only between the subfamilies but also between the individual subfamily members. This was proposed to lead to a functional segregation of the LC3 and GABARAP proteins. Indeed, the LC3 and GABARAP proteins were first identified in different compartments of human cells (microtubules for LC3 [123] and synaptic membranes for GABARAP [124], suggesting different functions for each subfamily. It could subsequently be shown that, upon starvation-induced autophagy, LC3-subfamily proteins are responsible for the elongation of the autophagosomal membranes, while GABARAPs are acting downstream, participating in the maturation and closure steps of the autophagosome formation [91]. Recent studies showed that only GABARAP-subfamily members are important for the activation of the phagophore-priming ULK1-ATG13-ATG101-FIP200 complex [125,126]. The centriolar satellites protein PCM1 binds unconjugated GABARAP and LC3C proteins to mediate their localization at the pericentriolar material and control autophagic degradation of centriolar satellites and GABARAPs [127,128]. Another example of the selective function of individual LC3/GABARAPs is the recruitment of LC3C to invading bacteria (*Salmonella typhimurium*) via the specific SAR, NDP52, important for autophagy-mediated restriction of bacterial growth. Depletion of both (NDP52 and LC3C) proteins is followed by an inability of the cell to defend the cytosol against invasion by *S. typhimurium*, while depletion of all other LC3/GABARAPs does not affect it [129]. Investigations of the molecular mechanisms behind these selective functions (e.g., linkage between residues at specific positions within LC3/GABARAP proteins and their functions) are only at their beginning.

In plants, the number of Atg8 orthologs varies from 1 in algae to 22 in angiosperms [68]; however, the diversity of plant Atg8 proteins could be significantly higher due to multiple gene duplications in order to adapt to various adverse conditions where Atg8 proteins play a crucial role [130]. The plant Atg8 proteins also reveal significant selectivity in interaction with their interaction partners, originated from the sequence difference between Atg8 homologs in different species [72]. Of note, all the key residues, participating in the HP1, HP2 [106] and UDS [122] are conserved, as well as the key lysine residues involved in regulating LIR binding: K49 and K51 in LC3A and LC3B (K46 and K49 in GABARAPs). The K49 performs a gatekeeper function, regulating the entrance of the aromatic residues of canonical LIRs into the HP1 [131]. Interestingly, K49A mutation significantly enhances binding of canonical LIRs to LC3B protein [131,132], while K51A decreased or abolished it. Drosophila Atg8a-LDS (K48A/Y49A) mutant flies exhibit strong accumulation of LIR-motif containing proteins like Ref(2)P and Kenny [133,134].

The N-terminal α-helices show significantly less conservation, which agrees with the hypothesis that these helices predetermine the selectivity of the interactions between Atg8 proteins and LIR motifs in SARs [118] and thus should be different in amino acid content for each individual family member. The few conserved residues within these α-helices participate either in folding of Atg8 proteins or in the formation of HP1. As expected, the loop regions are significantly less conserved, the relatively long loops L1, L2 and L3 show almost no identical or similar residues. Most conserved are regions of all β-strands, indicating their pivotal role in Atg8-protein folding and in the formation of HP1 and HP2.

## Unique features of GABARAP and LC3 subfamilies

The most significant consequence of the alignment shown in **Figure 2**, is the clear separation of all Atg8 proteins in LC3B and Atg8/GABARAP subtypes based on a few positions within their sequence [135]. This separation is also strongly supported by phylogenetic analyses of the nucleotide sequences of 36 Atg8 genes from 14 eukaryotic species (**Figure 1B**). The first conserved difference between LC3B and Atg8/GABARAP subtypes of proteins is the switch between the intramolecular electrostatic contacts for residues at positions 8 and 47 in Atg8/GABARAP (positions 10 and 50 in LC3B, respectively). For all Atg8/GABARAPs, position 8 is occupied by a negatively charged or polar residues which are able to play a role as hydrogen bond acceptors (Glu, Asp, Gln, Asn, Ser, and Thr), while position 47 is consistently used for electropositive residues (Lys and Arg), serving as the hydrogen bond donors. In contrast, in the LC3 subfamily there are electropositive residues (donors) at position 10 and electronegative residues (acceptors) at position 50. These residues come close to each other and form intramolecular hydrogen bonds or salt bridges to stabilize the Atg8/LC3/GABARAP structure and ensure a proper orientation of the N-terminal α-helical subdomain (**Figure 3E**). Importantly, these residues also form intermolecular contacts to residues in LIR motifs and, therefore, also contribute to the selectivity of Atg8 interactions with other proteins. Accordingly, phosphorylation of T50 by STK3, STK4, PKCζ and NEK9 [136,137], as well as introducing the phosphomimetic mutation T50E in LC3B strongly reduce both LC3B binding to several LIR-containing proteins, including FYCO1, and lipidation of LC3B [136]. FYCO1 is involved in directional transport of autophagosomes and blockade of T50 phosphorylation by STK4 decreases the starvation-induced perinuclear positioning of autophagosomes and their colocalization with lysosomes.

Another difference is consistently shorter long loops in Atg8 and GABARAPs. The loops between β-strands β1 and β2 (L1) and between β-strand β3 and α-helix α4 (L3) display no conservation; however, they undergo significant dynamic modulations in the free and LIR-bound forms of Atg8/LC3/GABARAPs, as was observed by NMR experiments [99,109]. Therefore, lack of one residue could in principle affect their dynamics and thus modulate selectivity to a specific LIR [138].

Although yeast Atg8 is more similar to the GABARAP subfamily in both sequence and structure, the extreme N-terminal region is more similar to the LC3 subfamily [74]. In Atg8 and LC3 subfamily proteins, the N-terminal arm is away from the ubiquitin fold and has a flexible open conformation, whereas in the GABARAP subfamily it has a closed conformation and tightly interacts with the ubiquitin fold. This structural difference is caused by the type of amino acid present at position 3 and 108 of Atg8: The GABARAP subfamily conserves Ala108 that forms hydrophobic interactions with Met1 and the aromatic residue (Phe/Trp) at position 3 in the N-terminal arm. Whereas in Atg8 and LC3 proteins position 3 is occupied by non-aromatic residues and position 108 is occupied by Val or Thr, impairing the interaction between the N-terminal arm and the ubiquitin fold. This structural difference is also observed between LGG-1 and LGG-2 in *C. elegans*, and may contribute to subfamily-specific functions.

## Selectivity determinants in LIR motifs

In species that express functionally distinct Atg8s, selectivity is regulated by subfamily or subset specific salt bridges, hydrogen bonds or hydrophobic interactions. Selectivity determinants in human Atg8 proteins are only partially characterized, but mutational analysis has shown that certain LIR core sequences possess an increased affinity to the GABARAP versus LC3 subfamily. This allowed the definition of a broad consensus for the GABARAP-interacting motif (GIM), conforming to the core sequence (W/F)-(V/I)-X-V [99]. A similar consensus sequence has not been made for LC3 binding, and there are also exceptions of GABARAP-selective LIR motifs (e.g. FIP200 and ATG14 LIR motifs) lacking a GIM. The residue in the +2 position in core LIR has a stronger effect on LC3 subfamily interactions than GABARAP subfamily interactions [100]. A combined use of mutational analysis, affinity measurements and X-ray crystallography revealed a tendency of the +2 residue to clash with LDS residues Q26, H27 and K30 in LC3B, while the corresponding residues in GABARAP (K24, Y25, R28) enables a more robust conformation that is less affected by the residue in the +2 position [100]. Y25 (invariant in all GABARAPs) participates frequently in the formation of intermolecular hydrogen bonds with positively charged or polar residues at position +1 of LIR motifs. The favorable conformation of Y25 is stabilized via cation-π interactions with a guanidinium moiety of invariant R28 (**Figure 3E**). The distinct conformation of Y25 and R28 makes the intermolecular hydrogen bonds more energetically favorable and thus increases the affinity of the LIR binding to the GABARAPs. In the LC3-subfamily, there are His or Phe residues at the position of GABARAPs Y25. Therefore, the favorable conformation of aromatic rings cannot be stabilized by cation-π interactions and effective intermolecular hydrogen bonds cannot form. How this can create selectivity is illustrated by the GABARAP selective LIR motifs of PCM1, ULK1 and FIP200 that are blocked in LC3 binding because they contain a Lys (PCM1) or Met (ULK1, FIP200) in position +2. For all these proteins, mutation of the +2 residue to Ile, Leu, Val or Phe resulted in LC3 binding, but no other residues gave LC3 binding [100]. Conversely, substitution of the +2 residue with Arg impairs LC3 binding of the AnkG LIR motif and renders it highly selective for GABARAPs [112].

The selectivity of a LIR motif can also be regulated by residues N-terminal to the core LIR. The above mentioned electropositive R10 and R11 residues (R16 and K17 in LC3C) on helix α1 are unique for the LC3 subfamily. They form important electrostatic interactions with acidic residues located in positions -2, -3 or -4 of a LIR motif [105,106,139]. Examples are the FYCO1 LIR (LC3 selective) that is stabilized by an electrostatic interaction formed between R10 in LC3B and D1277 in the -3 position of FYCO1 LIR [139], and the p62 LIR that is similarly stabilized by interactions formed between R10 and R11 in LC3B, respectively, with D337 and D338 in the -3 to -2 positions in the LIR of p62 [23,105,140]. However, acidic residues in position -1 and -2 are also crucial for GABARAP binding and the importance of N-terminal residues in creating selectivity is therefore restricted to the -3 and -4 positions [100].

More recent studies have revealed that residues C-terminal to the core motif can have a strong impact both on the selectivity and affinity of some LIR motifs. Many LIR motifs contain a negatively charged residue in the +7 position [141], and this enables the formation of an electrostatic interaction with the R70 residue in LC3B (R67 in GABARAP). Since this residue is conserved in all Atg8 proteins, the presence of a negative +7 residue does not affect the selectivity, but the affinity is normally increased. In some LIR motifs including the FYCO1 LIR [139,141], the +7 position marks the start of a short amphipathic α-helix that strengthens the interaction with Atg8 proteins. In LIR motifs of AnkG, AnkB, and FAM134B, this α-helix is extended resulting in very strong Atg8 binding affinity [112]. Studies of the LC3 selective LIR in FYCO1 and the GABARAP selective LIR in ALFY (autophagy-linked FYVE protein) identified position +5 as an important molecular selectivity determinant in LIR sequences [139,142]. In FYCO1, the D1285 residue in the +5 position provides specificity by binding to the H57 residue in LC3A or LC3B [139]. The corresponding residue in LC3C (Glu) or GABARAP (Asp) leads to charge repulsion and the selectivity is therefore directed towards LC3A and LC3B. The LIR motif in ALFY has a Tyr residue in the +5 position that clashes with the H57 residues in LC3A/B leading to repulsion of the interaction [142]. The Tyr residue instead binds to the corresponding Asp residue in GABARAPs (D54). Obviously, selectivity can be achieved in different ways, and the exact mechanism may vary between LIR motifs and often involve more than a single selectivity determinant. For the GABARAP selective LIR in ULK1, mutating the N-terminal -3 position (T to E), the +2 position in the core LIR (M to I) and the C-terminal +4 position (P to D) resulted in strong LC3B binding, and this illustrates the combined effect of three different selectivity determinants that all prevent the LC3A/B interaction [100].

Equally important as binding selectivity is the availability of individual Atg8s in a cellular context. The strong co-localization consistently seen between p62 and LC3B suggests that LC3B is a preferred interaction partner for p62, but we have also observed that most LIR proteins have low affinity for LC3B leading to less competition for binding this Atg8. A LIR motif occasionally overlaps with other binding motifs, and this similarly results in competition for binding. A relevant motif to mention here is FIR (FIP200 interacting region) that binds to the CLAW domain in RB1CC1/FIP200 [143]. FIR is identified in several proteins involved in autophagy, including p62 [143] , OPTN [144], CCPG1 [145], TBK1 adaptor proteins [146], and ATG16L1 [147,148]. The FIR consensus sequence is D/E/S/T-D/E/S/T-F/W/Y/I/L/V-X-X-I/L/V [149] that is very similar to the LIR consensus. The only important difference is that hydrophobic residues (I, L, V) are accepted in the aromatic Θ residue position, and there is also a stronger need for an acidic or phosphorylated residue in position -1 of a FIR. Because of this similarity, some LIR motifs (e. g. in p62 and OPTN) can function both as LIR and FIR. The dual LIR/FIR motif in OPTN (DS-FVEI) is activated by phosphorylation, and this strongly increases its binding efficiency for both Atg8s and RB1CC1/FIP200 [144]. The FIR2 motif in CCPG1 is similarly activated by phosphorylation and while its sequence (S-D-I-V-T-L) suggests it is not a LIR motif, binding experiments show that it can bind to mammalian Atg8s and RB1CC1/FIP200 [144]. The binding of LIR/FIR in p62 to RB1CC1/FIP200 may help in recruiting the core autophagy machinery, but the p62-RB1CC1/FIP200 interaction is probably excluded by the stronger interaction of polymeric p62 to lipidated Atg8s at the concave side of the phagophore [143]. There is an interesting similarity between the CLAW domain in RB1CC1/FIP200 and the domain in yeast Atg11 used for docking of SAR-cargo complexes to the PAS [149].

## LIR-LDS interaction is evolutionary conserved in *C. elegans* and *Drosophila*

Analysis of the LGG-1 and LGG-2 binding motifs in various interacting proteins in *C. elegans* showed that LGG-1 and LGG-2, similar to Atg8 family members in other systems, interact with the [W/F/Y]-x-x-[I/L/V] motif (LIR) [74]. Acidic residues (Glu or Asp) or Thr are preferred in the positions preceding the aromatic residue. The core sequence and surrounding residues of LIR confer binding specificity for LGG-1 or LGG-2. LGG-1 binds to “W”, “F” and “Y” type LIRs, while LGG-2 prefers Phe as the aromatic residue and Asp and Thr in the positions preceding the aromatic residue [74]. LGG-1 and LGG-2 also bind to substrates independent of the canonical LIR motif.

Crystal structures of LGG-1 and LGG-2 reveal that they exhibit a typical Atg8-family fold, which contains two N-terminal α-helixes (α1, α2) and a ubiquitin fold consisting of a four-stranded β-sheet and two α-helixes [74]. The N- and C-terminals of LGG-1 and LGG-2 display structural differences that result in differential binding to substrates. The C-terminal tails of Atg8 family members are flexible and divergent. The N-terminus of LGG-1, like GABARAP subfamily Atg8 proteins in mammals, exhibits a rigid closed conformation resulting from hydrophobic interactions between residues in the N-terminal arm and the ubiquitin fold (i.e. interactions formed by Met1 and Trp3 with Ala108 in LGG-1). The N-terminus of LGG-2, like the LC3 family and Atg8, is detached from the ubiquitin fold and thus adopts the open conformation. LGG-1 and LGG-2 possess the two hydrophobic pockets, HP1 and HP2, which recognize the aromatic [WFY]_0_ and [I/L/V]_+3_ core LIR residues, respectively, in their binding substrates. The HP1 and HP2 in LGG-1 and LGG-2 are structurally distinct. In the HP1 site in LGG-1, a key Phe residue is adjacent to a Gly residue, which allows rotation of the Phe benzene ring. Thus, HP1 shows plasticity in accommodating divergent aromatic residues. In the HP1 of LGG-2, the conformation of the corresponding Phe is restricted by juxtaposition with a bulky Val residue. The HP2 in LGG-1 and LGG-2, which differ in size and shape, show different preference for Leu and Val at the “+3” position in the LIR [74]. The N-termini of LGG-1 and LGG-2 contribute to binding with the residues preceding the aromatic residue in LIR. For example, the Arg residues at the N-termini form electrostatic interactions with the acidic residues adjacent to LIR [74]. Therefore, the structural differences in the HP1, the HP2, and the N-termini of LGG-1 and LGG-2 determine their binding specificity for interacting proteins.

In *Drosophila*, a conservation of F3 and A108 residues in Atg8a suggests that this protein has a closed conformation typical for metazoan GABARAP subfamily. However, these residues are not conserved in Atg8b (**Figure 2**). A range of LIR-motif containing proteins found in mammals also have functional equivalents in *Drosophila*, many of which interact with Atg8a via the aid of a LIR motif. As with many other lower complexity model organisms, often their relative ease of study allows for early and cost-effective investigations that can provide helpful insight to inform research into higher complexity models [150]. Recently, a high-throughput yeast 2-hybrid (Y2H) screening identified several Atg8a-interacting proteins in *Drosophila* [151]. These proteins can be classified in 3 groups: 1) proteins which have already been experimentally verified to bind Atg8a, such as Atg1, DOR, Ref(2)P and Kenny, 2) proteins for which their mammalian homologs interact with Atg8-family members, like Ank2, Atg4, and Nedd4 and 3) several novel Atg8a-interacting proteins, such as trc/STK38 and Tak1 [151]. Of note, upon sequence analysis using the LIR-prediction software iLIR, all proteins of this Y2H list are found to possess at least one, often several, candidate LIR motif(s) within positions that overlap with the predicted Atg8a-interaction region mapped by the Y2H screen for each hit [151]. In accordance with the typical LIR peptide structure, most of the characterized LIR motifs for the known Atg8a-interactors in *Drosophila*, are hexapeptide sequences that bear the typical the typical Θ_0_-X_1_-X_2_-Γ_3_pattern, (where Θ is an aromatic (W/F/Y) and Γ is a hydrophobic (L/I/V) residue) at their core [152].

## The need for multivalent LIR-LDS interactions in docking of cargos to the phagophore in selective autophagy

Only a subset of Atg8 binding proteins work as SARs involved in selective autophagy, but these SARs are extremely important for this process since they are responsible for the docking of a selected cargo to the phagophore membrane. Presumably, a selected cargo must have a certain size to enable growth of a phagophore on its surface. In addition, the selectivity is driven by a tight binding between the SAR-cargo complex and Atg8 proteins lipidated to the phagophore membrane. Several studies have shown that autophagosome formation is possible without Atg8 proteins, but the efficiency is low and the GABARAP subfamily is required for the closure step [101,102]. In addition, SAR-cargo complexes are only randomly engulfed in the absence of Atg8 proteins and there is no selectivity in the process. Martens used the term exclusive autophagy to describe the growth of a phagophore on a selected cargo [153] emphasizing the need for multiple interactions between a SAR and Atg8 proteins in selective autophagy. Multivalency can be achieved in different ways, but we will here mention two different strategies. p62 is a polymeric protein that is presumably attached as a polymer to the phagophore membrane [154-156]. Atg19 is however monomeric, but it contains several cryptic LIR sequences that are activated if the main LIR in Atg19 is bound to an Atg8 [153].

## Non-canonical LIR motifs: Half-LIRs and α-helical LIRs

An increasing number of noncanonical LIR sequences either lack the aromatic Θ_0_ residue or the hydrophobic Γ_+3_ residue and can be seen as “half-LIRs”. The first non-canonical LIR to be described was the LIR of NDP52 (I^333^LVV) that lacks the aromatic Θ residue and therefore does not engage HP1 [129]. The side chain of the Ile residue in Θ position is too short to occupy the aromatic pocket, but the LIR of NDP52 binds selectively and strongly to LC3C (therefore named CLIR). The LVV motif in CLIR forms compensating hydrophobic interactions with residues Lys32, Phe33, Leu64, Phe69 in LC3C, and these residues are misaligned or absent in other human Atg8s explaining the selectivity for LC3C [129]. The importance of the missing aromatic Θ residue is illustrated by that mutation of the NDP52 core motif from ILVV to WLVV results in binding to all human Atg8s [129]. Other reported non-canonical LIR sequences have no hydrophobic Γ residue and therefore do not engage HP2. Examples are the LIRs in TRIM5α (DW^196^E) [157,158] and BCL-2 (EW^30^D) [159], which both have a Trp residue docked into HP1. Another non-canonical feature of the LIR in TRIM5α is that it is helical and located in a coiled-coil domain [157]. In a canonical LIR-LDS interaction the LIR is kept within the structure of a β-sheet. Helical LIR motifs have higher structural flexibility, and the LIR consensus sequence is less rigid. This may result in a docking of non-consensus residues into pockets HP1 and HP2, and the distance separating the two residues may also vary. In the structure formed between the coiled-coil domain of TRIM5α and LC3B, no residue is docked into HP2, but Gln203 is located on the edge of the HP2 pocket and replacing it with a smaller hydrophobic residue gave increased binding affinity. The TRIM5α LIR has a rather weak binding affinity, but the affinity is strongly increased by dimerization of TRIM5β [157]. Another type of non-canonical LIR motif is found in UBA5. This motif binds to the GABARAP subfamily, but also to the ubiquitin-like modifier UFM1 [160]. The binding of this LIR motif results in the formation of an intermolecular β-sheet, but three hydrophobic pockets (not the usual two) are formed at the interphase on GABARAP. Rearrangements of GABARAP residues K46 and K/R47 results in the formation of an additional pocket termed HP0 (**Figure 3C**). The LIR in UBA5 (W^341^GIELV) has W^341^ docked into HP0, while the canonical HP1 and HP2 sites are occupied by I^343^ and L^346^ [138]. The GABARAP selectivity of this LIR motif strongly depends on the above-mentioned K/R47 residue unique for the GABARAP subfamily (D50 in LC3B). A few proteins, including the RavZ protein of the intracellular pathogen *Legionella pneumophila* [161], human FNIP1 [162] and human ATG3 [163], have a LIR embedded in a β-sheet. The core LIR of human Atg3 (W^107^VDT) is unusual by having a Thr instead of the usual Ile, Leu or Val residues in position +3, but W^107^ is docked into HP1 and T^110^ into HP2 as in canonical interactions. However, while the interaction with GABARAP is completely blocked by a mutation of W^107^, mutation of T^110^ does not affect the interaction [163].

## Regulation of LIR-LDS interactions by phosphorylation of LIR motifs

**Figure 4.**
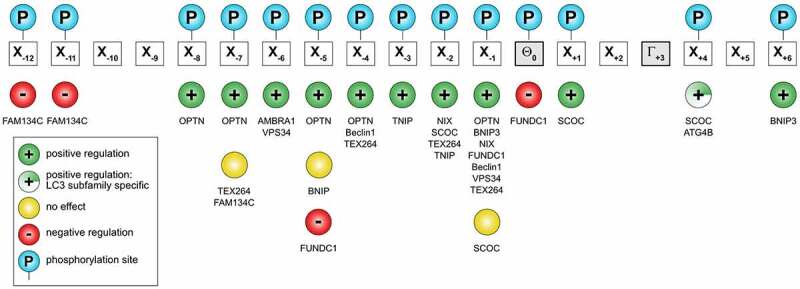
*Distribution of known phosphorylation sites on LIR motifs on some mammalian Atg8-interacting proteins*. Known positive (green circle with plus sign) or negative effects (red circle with minus sign) of phosphorylation (blue circle) of a particular residue relative to binding to Atg8s indicated. Yellow circle and half-filled green circle with plus sign indicate no regulation of ATG8 binding and positive effect specific to LC3 subfamily binding, respectively. The actual proteins are shown below each site. See also Table 1.

Phosphorylation of residues within the N-terminal flanking region of the core LIR (mostly preceding the aromatic residue at positions -1 or -2) may enhance the affinity of the SAR:Atg8/LC3/GABARAP binding [109,132,164-170], and serves as a key autophagy regulator in corresponding types of selective autophagy. LIR sequences usually contain residues that can be phosphorylated (Ser/Thr) and it is seen for a large number of investigated proteins that phosphomimetic mutations increase their affinity to Atg8/LC3/GABARAPs (**Figure 4**). The strongest effect is usually seen when a negatively charged or phosphorylated residue is located at positions -2 or -1 preceding Θ, but also more distant residues (positions -8 to -3) may affect the affinity of a LIR-LDS interaction [168,171]. For the optineurin (OPTN) LIR, phosphorylation of individual Ser residues up to position -8 still increases its affinity to LC3B [109]. IKKα-mediated phosphorylation of AMBRA1 S1014 at position -6 promotes AMBRA1’s binding to LC3 and GABARAP (*in vitro* and *in vivo*) and serves as a positive regulator of AMBRA1-mediated mitophagy [165]. In some cases, like for FUNDC1, direct phosphorylation of the Tyr residue at the LIR Θ position leads to a weakening of the LIR:LC3/GABARAP binding affinity [166].

Post-translational modification of a LIR motif is normally phosphorylation, and around 25% of LIR motifs has a Ser or Thr residue in the critical -1 position [152]. It is convenient to distinguish between LIR motifs that depend on post-translational modification and those that do not. Known regulatory phosphorylation sites within LIR domains of mammalian proteins are shown in **Table 1**. There is so far no direct evidence that LIR motifs in p62 or NBR1 are regulated by post-translational modification. This correlates with a constitutive degradation of these soluble SARs by selective autophagy [172]. Post-translational activation of LIR motifs was first shown for the soluble SAR OPTN [167]. The involvement of OPTN in selective autophagy (e. g. xenophagy, mitophagy, aggrephagy) depends on TBK1 mediated phosphorylation of S177 in its LIR motif (position -1) [167]. Unlike other SARs, OPTN is degraded by the proteasome if not involved in selective autophagy [173], and this correlates with the need for activation of its LIR motif. Organelle resident SARs sit on the surface of structures that are normally not degraded, and it is obvious that their functions in selective autophagy needs to be tightly regulated. Indeed, early studies on mitophagy receptors BNIP3L/NIX, BNIP3 and FUNDC1 revealed that activation by phosphorylation is a common mechanism for regulating LIR motifs in mitophagy receptors [174]. The LIR in BNIP3 is activated by phosphorylation of residues Ser17 and Ser24 flanking the core motif in positions -1 and +6 [170], LIR in NBIP3/NIX is activated by phosphorylation of Ser34 in position -1 [132], while LIR in FUNDC1 is regulated both positively and negatively by phosphorylation. The function of FUNDC1 in mitophagy is induced by hypoxia, and its LIR motif (ESDDDSYEVL) is kept inactive under normoxia by the phosphorylation of two residues: i. e. Ser13 in position -5 [175] and Tyr18 in position 0 [176]. Hypoxia results in dephosphorylation of both these sites by PGAM5 and recruited ULK1 further activates the LIR motif by phosphorylating Ser17 in the -1 position [175,177]. Phosphorylation of Tyr18 is a good example on how Y-type LIR motifs can be inactivated by phosphorylation of the essential aromatic residue. The negative regulation seen for the Ser13 phosphorylation may reflect its location in the LIR motif that is more distant to the core motif (-5) than seen for most negatively charged residues or phosphorylation sites having a positive effect on the LIR-LDS interaction. Ser13 in FUNDC1 is phosphorylated by CK2 [175]. The same kinase was recently shown to phosphorylate three Ser residues in positions -12, -11 and -7 in the LIR motif of reticulophagy receptor FAM134C, thereby inhibiting the binding of FAM134C to Atg8s under fed conditions [178]. The authors explained the inhibitory effect by the notion that phosphorylated residues more distal to the core LIR interact with a region in LC3B with a neutral charge and are therefore disfavored.

**Table 1:** Phosphorylation sites within LIR domains of mammalian proteins

Protein	LIR domain	Residue (kinase/phosphatase, if known)	References
AMBRA1	EALN**S**GVEYY**WDQL**NETVFTVHSN	S1014 (IKKβ)	[165]
ATG4B	ERFFDSEDED**FEILS**L	S249	[94,197]
Beclin 1	RMM**S**TE**S**AN**SFTLI**GEASDGGTME	S90 (MAPKAPK2, MAPKAPK3, DAPK/PPP2); S93, S96 (AMPK)	[96,198-200]
BNIP3	GMQEE**S**LQG**SWVEL**HF**S**NNGNGGS	S13; S17 (ULK1), S24	[170,201]
NIX/BNIP3L	LPPPAGLN**SSWVEL**PMNSSNGNDN	S34; S35 (ULK1)	[132,201]
FAM134C	**SS**DLD**T**DAEGDD**FEEL**DQSELSQLDP	S435, S436, T440 (CK2)	[178]
FUNDC1	PQDYE**S**DDD**SYEVL**DLTEYARRHQ	S13 (CK2, PGAM5), S17 (ULK1), Y18 (PGAM5)	[175,176,202]
OPTN	LN**SS**G**SS**ED**SFVEI**RMAEGEAEGS	S170, S171, S173, S174; S177 (TBK1)	[109,167]
SCOC	SRKEEEED**STFTNIS**LADDIDHSS	S12, T13, T15, S18	[168]
TEX264	YSE**S**GA**S**G**SSFEEL**DLEGEGPLGE	S266, S269, S271, S272 (CK2)	[164]
TNIP1	KPPSSGT**SS**E**FEVV**TPEEQNSPES	S122, S123 (TBK1)	[169],[203]

A common way of testing the effect of phosphorylation on Atg8 binding is the use of phosphomimic mutations. However, this type of mutations may not always mimic the effect of phosphorylation. A recent study on the reticulophagy receptor TEX264 reported that phosphorylation of two Ser residues adjacent to the core LIR (SSFEEL), again by CK2, is essential for its interaction with Atg8s and induction of reticulophagy [164]. In this case, phosphomimic mutations had no such effect, and crystal structures of GABARAP in complex with TEX264 LIR peptides revealed important structural differences in complexes formed with phosphorylated and phosphomimic mutated LIR peptides. The main difference was the presence of four specific hydrogen bonds with the phosphorylated peptide that were not formed with the mutated peptide that instead gave nonspecific salt bridges [164].

## Interactions involving the UDS

A number of Atg8 binding proteins interact in a LIR-LDS independent manner [179]. A search for other binding mechanisms led to the identification of ubiquitin interacting motif (UIM)-like sequences as candidates for a new type of Atg8 interacting motif. The UIM-like sequences were shown by yeast two hybrid assays and site directed mutagenesis to bind to a specific patch on the Atg8s which was termed the UIM-docking site (UDS) [122]. The UDS was defined as a patch of four residues that are highly conserved in evolution and has the core consensus sequenceψ-F-ψ-ω/T [122]. The UDS is localized on the opposite surface of the Atg8s relative to LDS and relatively close to the C-terminal Gly residue. This means that the UDS points towards the membrane when Atg8 is lipidated [37], and it is therefore not fully understood how a UIM motif can interact with the lipidated form. Importantly, so far there are no structural data to support the notion of UIM-like sequences binding to the UDS. However, structural data exist for the interaction of ATG4B [93] and Hfl1 [42] with the UDS region, but no UIM-like sequences has been identified in these proteins. A main question is if the UIM-UDS interaction is restricted to unlipidated Atg8s or if it is also possible for Atg8s that are lipidated to a membrane. Further studies and structural evidence is needed before a conclusion can be made. Interestingly, both the LDS and UDS regions were marked as important already in 2006, when it was shown that sites involving Y49 and L50 on one (LDS) side, and F77 and F79 on the opposite (UDS) side, of yeast Atg8 were essential sites for autophagy [180].

## Composite interactions involving both LDS and UDS

The non-canonical CLIR motif of NDP52 (ILVV) is evolutionary conserved in the paralogs TAX1BP1 and CALCOCO1, but the selectivity for LC3C is not conserved and the non-canonical motif in CALCOCO1 (LLVV) has preference for the GABARAP subfamily [181]. The sequence adjacent to core LIR differs in the three paralogs and the CLIR of NDP52 contains more negatively charged residues. The binding of the isolated LIR motif of CALCOCO1 is very weak even for the GABARAP subfamily. However, the interaction is strongly increased by dimerization of CALCOCO1 and further increased by an additional weak interaction with the hydrophobic UDS patch including the essential Phe residue (F^77^ in GABARAP) [181]. No UIM-like sequence was identified in CALCOCO1 and the UDS interacting motif mapped to residues 615-653 was therefore named UIR (UDS interacting region). Yeast vacuole membrane protein Hfl1 is another example of a protein where a weak non-canonical LIR motif is supported by an additional interaction with UDS [42]. Unlike UIR in CALCOCO1 that is located distal to LIR, the UDS interacting region in Hfl1 is located adjacent to LIR. The noncanonical LIR in Hfl1 is helical. The spacers separating the aromatic Θ and hydrophobic Γ residues are extended in the LIRs of ScHfl1 (W^371^xxxI) and SpHfl1 (F^388^xxxxxxxxxY), and the latter has a Tyr residue docked into HP2 [42]. In addition, a Tyr residue adjacent to the non-canonical LIR Y^387^ in ScHfl1) interacts with a so-called Y-site in UDS of Atg8 and this increases the strength of the interaction (**Figure 3D**). These two examples demonstrate that simultaneous interaction with LDS and UDS is possible, but the frequency is not known since the weakest interaction is easily overlooked. It should be noted that yeast Hfl1 interacts with unlipidated Atg8 for its function, and it is not known if UDS is exposed and can facilitate binding if Atg8 is lipidated to a membrane. Another interesting example of a protein binding LDS and UDS is Atg4. There is no crystal structure of full-length Atg4, but structures of human LC3B bound to the catalytic domain of Atg4B revealed an interaction with UDS and residues surrounding UDS [93]. In addition, the C-terminal tail of Atg4B contains an evolutionary conserved and canonical LIR motif that binds strongly to LDS, and functional studies of this LIR has been performed in yeast and mammals [94,182]. How the UDS and LDS interactions are utilized in Atg8 processing or delipidation is not clear. However, in mammals a highly selective role of Atg4B is stabilizing a free pool of unlipidated GABARAP and GABARAPL1 and this depends on a strong interaction enabled by using both interaction surfaces [94].

## Interactions involving the N-terminal arm

The canonical LIR-LDS interaction is dependent on both the N-terminal arm domain (amino acids 1–28) and the ubiquitin core (amino acids 30–120) of LC3B [23,140,172]. However, there are several reports of interactions with Atg8 family proteins that do not involve the LDS but rather the N-terminal arm. Hence, *Drosophila* KEAP1 binds to the N-terminal 71 amino acids of *Drosophila* Atg8a. The LDS mutant K48A/Y49A does not affect binding. However, the N-terminal arm alone is not sufficient as amino acids 1-26 does not bind to *Drosophila* KEAP1, neither does a piece from amino acid 26 to 71 showing that the region 1-71 of Atg8 is required [183]. The same interaction pattern was seen between *Drosophila* YL-1, a component of a nuclear acetyltransferase complex, and Atg8a [15]. When oncogene induced senescence is triggered by expression of activated Ras, Lamin B1 interacts with nuclear LC3B and the complex is exported out of the nucleus to be degraded by autophagy in the cytoplasm. The N-terminal arm of LC3B with amino acids 1-28 is sufficient to bind to human Lamin B1 with R10 and R11 being essential for binding [13]. Upon lysosomal damage, GABARAP binds to the core stress granule proteins NUFIP2 and G3BP1 and recruits them to damaged lysosomes where NUFIP2 helps to inactivate mTOR via the Ragulator-RagA/B complex. Both NUFIP2 and G3BP1 bind to the N-terminal arm of GABARAP and mutants of the LDS or UDS do not affect the binding [12]. Whereas NUFIP2 also interacts with the ubiquitin core of GABARAP, G3BP1 only binds to the N-terminal arm. Two regions of NUFIP2 bind to GABARAP whereas it is the N-terminal NTF2L domain of G3BP1 which binds to the N-terminal 1-26 amino acids of GABARAP. Recognition of damaged mitochondria is required for cellular health. Hence, redistribution of cardiolipin from the inner to the outer mitochondrial membrane acts as an “eat-me” signal for mitophagy in neuronal cells. Cardiolipin binds directly to LC3 with R10 and R11 in the N-terminal arm being essential for the binding and the biological response [119]. We will surely see more examples in the future of interactions depending on the N-terminal arm of Atg8s. However, so far we sorely miss structural data on these type of interactions.

## Prediction of LIR motifs in proteins

Sequence motif-based prediction of LIR motifs in proteins employing regular expression pattern was pioneered by the iLIR software tool [184]. Subsequently, the iLIR web resource for LIR-containing proteins in *Arabidopsis, C. elegans*, chicken, human, mouse rat, zebrafish and *S. cerevisiae* was established, followed by the iLIR@viral web resource for LIR-containing viral proteins [185,186]. The most recent development is the LIRcentral, a web accessible database (LIRcentral; https://lircentral.eu) that contains information about literature-validated LIR-motifs and displays them along with features annotated in UniProt. By cross-referencing protein entities to UniProt entries, LIRcentral enables seamless data integration with other resources [187]. It is possible to improve on the iLIR and other regular expression based sequence prediction methods by manually curating the candidate hits by excluding motifs that contain residues within the core LIR that usually are inhibitory to binding such as glycine (G) and proline (P) that affect the secondary structure, and the basic lysine (K) and arginine (R) residues that can mediate charge repulsion, due to the basic residues surrounding the two hydrophobic pockets of the LDS [9,95].

However, iLIR, and other sequence motif-based prediction tools, cannot predict any of the non-canonical LIR sequences and it is challenging to predict half-LIRs as well, particularly in long sequences. An exciting new development that holds promise of more precise LIR predictions is the use of the AlphaFold2-multimer artificial intelligence-based protein modelling tool. Ibrahim et al. combined protein modelling using AF2-multimer with phylogenetic analysis of protein sequences and protein-protein interaction assays [188]. Strikingly, the AF2- multimer enabled high accuracy prediction of canonical and also some non-canonical LIR motifs. When more non-canonical LIR/LDS structures are known the AF2 multimer predictions of such interactions will become more reliable.

## Binding motifs anticipated to be found in future investigations

The LC3/GABARAP interactome in human contains ~400 potential candidates under basal autophagy conditions [179]. Only a small fraction of these proteins were validated and characterized as LC3/GABARAP binders, while validation of the rest and/or discovery of new candidates is complicated by the fact that researchers are looking mostly for the conventional and well-characterized canonical LIR motifs as the interaction determinant. This strategy, however, will not be sufficient in the light of the growing examples of unusual mechanism for interactions of Atg8/LC3/GABARAPs their partners. Below we summarize suggested (but not investigated so far) structural motifs which could be implicated in these interactions and may serve as a starting point for new investigations.

## I. Antiparallel LIR motifs

**Figure 5.**
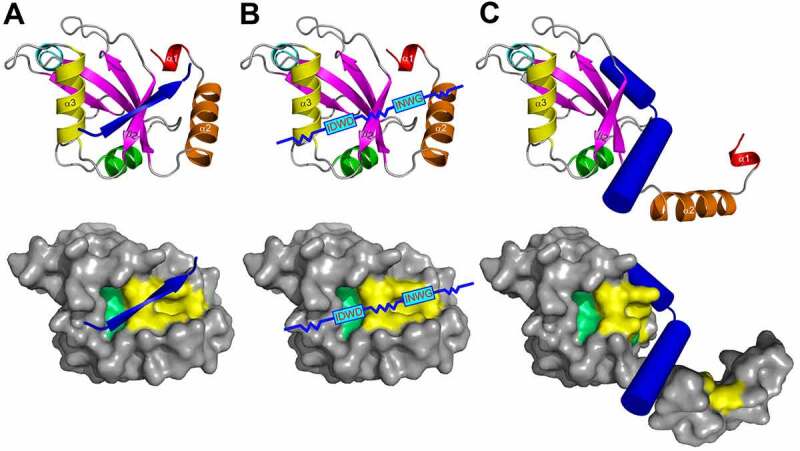
*Emerging types of Atg8/LC3/GABARAP interacting motifs and elements*: the antiparallel β-strand (**A**), the shuffled LIR motifs (**B**), and displacing α-helical structure (**C**). The interacting elements shown as blue arrows or cylinders on LC3B ribbon diagram (top) and on LC3B surface (bottom, with HP1 and HP2 indicated). The shuffled LIRs motifs are given as sequences in the blue boxes, their structural mechanism with Atg8/LC3/GABARAPs is not known, however, preliminary data indicate their LDS-guided binding.

For all the canonical and non-canonical LIR sequences identified to date, the orientation of the extended β-stranded conformation of the LIR peptide is parallel to the β-strand β2 in Atg8/LC3/GABARAP. The only exception reported so far is the structure of an artificial peptide called K1 in complex with GABARAP [189]. Therefore, one can predict that antiparallel β-stranded linear peptides with a reverse order of residues for Θ and Γ positions and with a corresponding C-terminal track of negatively charged residues after the core aromatic residue (N’-Γ-X-X-Θ-X^–^X^–^X^−^ instead of N’-X^–^X^–^X^−^Θ-X-X-Γ) could efficiently bind Atg8/LC3/GABARAP proteins [135](**Figure 5A**).

### II. LIR motifs with non-canonical sequences

Considering other non-canonical linear LIR sequences (cLIR in NDP52 and LIR/UFIM in UBA5), one can predict existence of a high number of LIR-like sequences representing this category. The attempt to generate (by a phage display) high affinity and highly selective synthetic peptides capable of binding individual members of LC3 and GABARAP subfamilies in human cells led to generation of a number of sensor molecules; however, they all seem to contain canonical LIR motifs [190]. The hypothetical motifs could be organized in various ways (displaying sequence complexity and using “through-space” binding modes) to facilitate effective and specific binding of Atg8-family proteins. For example, the evolutionary conserved ERphagy receptor C53 (called CDK5RAP3 in humans) binds plant and mammalian Atg8s via so-called shuffled AIM/LIR located within the intrinsic disordered central region of C53. These are versatile binding sites allowing both binding to Atg8s and to UFM1 and composed by shuffled AIM sequences (like IDWD, **Figure 5B**). It is not clear, where and how exactly these shuffled AIMs interact with Atg8s. Hence, structural aspects of these interactions should be investigated in detail. But, as shown by NMR studies, the binding is different to Atg8s and UFM1. C53-mediated autophagy clears toxic incomplete polypeptides from translation generated under stress conditions. At normal conditions, C53 is inactive as an ERphagy receptor because it binds UFM1. Upon stress it is displaced by Atg8s activating ERphagy [191,192].

#### III. Binding motifs that do not use the LDS/UDS sites on the Atg8/LC3/GABARAP surface

It was proposed [135], that the α-helical subdomain can be displaced from the ubiquitin core of Atg8/LC3/GABARAP proteins by another α-helical structure containing a combination of residues, which are more favorable for the binding of the ubiquitin core of a particular Atg8/LC3/GABARAP protein (**Figure 5C**). More aggressive conditions, which appear in close proximity to membranes or in cellular compartments with critical pH values, might facilitate the displacement. In this case, the amino acid content of the displacing α-helices could significantly differ from that for displaced helices α1 and α2, leading to the HP1 modulation in shape and dynamics.

The N-terminal α-helical subdomain in Atg8/LC3/GABARAP proteins is a key evolutionary addition to the core ubiquitin-like fold to separate structurally and functionally the autophagy modifiers from any other UBLs. The α-helices show a significant conformational exchange [109,115,125,193] and could potentially be separated from the ubiquitin core as the truncated LC3B and GABARAPL2 proteins were still able to perform some functions, like membrane fusion [118]. The first α-helix in LC3B and GABARAPL2 was successfully swapped to emphasize their role in p62/SQSTM1 recognition [194]. Moreover, it was shown that GABARAPL1 being truncated N-terminally for the α-helical subdomain and the β-strand β1 could still recognize and bind a number of cognate receptors and proteins (γ2 subunit of GABA_A_ receptor [136], human κ opioid receptor [195], gephyrin [196]).

## Conclusions - future perspectives

Since the discovery of the LIR/AIM motifs a large number of Atg8-interacting proteins have been identified in all model organisms used for autophagy studies as well as in humans. The approach of identifying Atg8-interacting proteins has had a major impact on autophagy research particularly in studies of selective autophagy. We have learned a lot on the nature of the LIR-LDS interactions from structural studies combined with mutagenesis and protein-protein interaction analyses. New tools for predicting LIR-Atg8 interactions have emerged along with databases of LIR motifs and LIR-containing proteins in many species. This will aid in a more rapid discovery of remaining unidentified LIR-containing proteins, also those containing non-canonical motifs. The power in combining artificial intelligence aided 3D modeling and docking with evolutionary conservation and binding analyses using mutants in the LIR motifs and in the LDS will aid both in discovery and validation of new LIR-containing proteins. Other interaction modes, are beginning to be described. Hence, it will be important to obtain structural data on interactions involving the UDS and the N-terminal arm to understand these better. This will likely aid us in identifying new Atg8-interacting proteins that use these interaction surfaces. We will likely also discover more combined motifs as already exemplified by the UFIM and Atg8 interaction motifs found in UBA5 and C53.
